# The World Health Organization’s public health intelligence activities during the COVID-19 pandemic response, December 2019 to December 2021

**DOI:** 10.2807/1560-7917.ES.2022.27.49.2200142

**Published:** 2022-12-08

**Authors:** Eri Togami, Bridget Griffith, Mostafa Mahran, Ingrid H Nezu, Bernadette B Mirembe, Kaja Kaasik-Aaslav, Lidia Alexandrova-Ezerska, Amarnath Babu, Tika Ram Sedai, Masaya Kato, Heidi Abbas, Mahmoud Sadek, Pierre Nabeth, Lauren E. MacDonald, Lucía Hernández-García, Jeffrey Pires, Stefany Ildefonso, Mary Stephen, Theresa Min-Hyung Lee, Benido Impouma, Tamano Matsui, Sangjun Moon, Manilay Phenxay, Viema Biaukula, Ariuntuya Ochirpurev, Johannes Schnitzler, Julie Fontaine, Irena Djordjevic, Hannah Brindle, Jessica Kolmer, Martina McMenamin, Emilie Peron, Zyleen Kassamali, Blanche Greene-Cramer, Esther Hamblion, Philip Abdelmalik, Boris I Pavlin, Abdi Rahman Mahamud, Oliver Morgan

**Affiliations:** 1 Health Emergencies Programme, World Health Organization (WHO) Headquarters, Geneva, Switzerland; 2 Health Emergencies Programme, WHO Regional office for South-East Asia, New Delhi, India; 3 Health Emergencies Programme, WHO Regional office for the Eastern Mediterranean, Cairo, Egypt; 4 Health Emergencies Programme, WHO Regional office for Europe, Copenhagen, Denmark; 5 Health Emergencies Programme, Pan American Health Organization/WHO Regional Office for the Americas, Washington DC, United States of America; 6 On behalf of the Health Emergency Information and Risk Management (HIM) team, WHO Regional Office for African Region, Brazzaville, Republic of the Congo, whose members are acknowledged at the end of the article; 7 Health Emergencies Programme, WHO Regional Office for the Western Pacific, Manila, Philippines

**Keywords:** event-based surveillance, COVID-19, public health intelligence, SARS-CoV-2, epidemiology, surveillance

## Abstract

The coronavirus disease (COVID-19) presented a unique opportunity for the World Health Organization (WHO) to utilise public health intelligence (PHI) for pandemic response. WHO systematically captured mainly unstructured information (e.g. media articles, listservs, community-based reporting) for public health intelligence purposes. WHO used the Epidemic Intelligence from Open Sources (EIOS) system as one of the information sources for PHI. The processes and scope for PHI were adapted as the pandemic evolved and tailored to regional response needs. During the early months of the pandemic, media monitoring complemented official case and death reporting through the International Health Regulations mechanism and triggered alerts. As the pandemic evolved, PHI activities prioritised identifying epidemiological trends to supplement the information available through indicator-based surveillance reported to WHO. The PHI scope evolved over time to include vaccine introduction, emergence of severe acute respiratory syndrome coronavirus 2 (SARS-CoV-2) variants, unusual clinical manifestations and upsurges in cases, hospitalisation and death incidences at subnational levels. Triaging the unprecedented high volume of information challenged surveillance activities but was managed by collaborative information sharing. The evolution of PHI activities using multiple sources in WHO’s response to the COVID-19 pandemic illustrates the future directions in which PHI methodologies could be developed and used.

## Introduction

The World Health Organization (WHO) uses public health intelligence (PHI) as one element of its approach to improve population health. A PHI approach encompasses the detection, verification, risk assessment and investigation of events that pose a potential risk to human health, and communicating this information for effective decision making and action [1-3]. Public health intelligence requires the systematic synthesis of different types and sources of information, which are gathered through indicator-based surveillance (IBS), which includes counts of patient counts, cases, or laboratory diagnoses, and event-based surveillance (EBS) which comprises predominantly unstructured information from other sources, including from non-health sectors, that are used for public health surveillance purposes, such as media articles, listservs and community-based reporting [4-6]. We describe how the PHI activities for the coronavirus disease (COVID-19) contributed to WHO’s COVID-19 response, the evolution of PHI activities from December 2019 to December 2021, and lessons for the future.

## The role of public health intelligence in the WHO response to the COVID-19 pandemic

Event-based surveillance involves the detection, triage and verification of new public health threats and changes in ongoing events. Signals, defined as raw data or information with potential acute human health risk [7], are detected, risks-assessed, documented and followed daily, based on predefined criteria.

**Figure 1:**
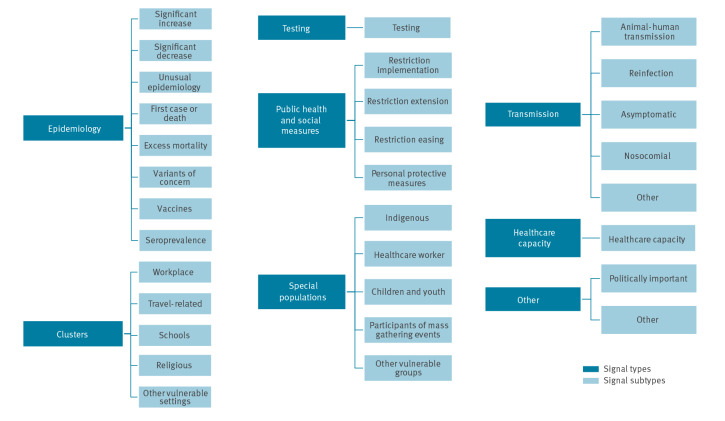
Categorisation of COVID-19 Public Health Intelligence activities, as of December 2021^a^

^a^ These are the types and subtypes of categories detected by the Global team within the WHO headquarters COVID-19 Incident Management Support Team, and do not represent all types of PHI information detected by WHO. The scope of signals is adapted based on the evolution of the pandemic, and additional signal types may be monitored and detected based on surveillance and response needs.

One of the tools used by WHO for PHI to monitor all hazards, including COVID-19, is the Epidemic Intelligence from Open Sources (EIOS) system [8]. Here, publicly available online information such as media articles, government websites and social media are monitored. Between December 2019 and December 2021, the public health intelligence generated through EIOS and other sources provided important information for epidemiological interpretation and risk assessment to guide WHO’s COVID-19 response (Figure 1).

With the emergence of COVID-19, additional event-specific criteria were established and adapted over time to reflect the pandemic’s evolution and the evolving response needs. These additional criteria were determined as a result of a discussion between a team of WHO epidemiologists who took into account surveillance objectives and scope, the epidemiological context in which the pandemic took place, and available human resources. The criteria were continuously reassessed every several months, or when the characteristics of the pandemic changed. COVID-19 PHI provided a more complete understanding of disease dynamics rather than relying on IBS alone.

## Epidemic Intelligence from Open Sources system

The EIOS system was one component key for WHO PHI activities during the COVID-19 pandemic. EIOS is an adaptable, user-oriented and constantly evolving web-based system designed to support, augment and accelerate PHI activities. It is used by communities and organisations from national to international levels [9-11]. The EIOS system is one of the tool to monitor open-source information in emergencies. PHI teams across WHO offices use the system to detect unusual or unexpected signals at various geographic levels. The system gathers pieces of information in multiple languages on a near real-time basis from over 13,000 sources. To improve geographical and thematic coverage, information sources are continuously reviewed and added, in collaboration with user communities such as national health authorities and partner organisations. Individuals and user communities can tailor the system by defining selection criteria based on parameters which include potential health threats, time, country, locations, sources and language. Furthermore, users can pin, flag and export selected articles to collaborate and communicate with stakeholders according to their data sharing needs. Users can also provide input to add new categories of interest, modify the category definitions and add sources as emergencies evolve.

## Evolution of public health intelligence activities during different stages of the COVID-19 pandemic

Prior to the detection of COVID-19, the WHO was conducting standardised EBS daily, employing an all-hazards approach and using official government reports, EIOS and other sources of information. Building on this established mechanism at the onset of the pandemic, the WHO intensified EBS to track initial cases and deaths from COVID-19 while an IBS system was being established to complement International Health Regulations (2005) reporting mechanisms. Due to the volume of information, the number of analysts dedicated to screening for COVID-19-related events was increased. In addition, a separate set of filter criteria was created within the EIOS system exclusively for monitoring information related to COVID-19, which reduced the volume of articles on the routine EBS boards. Systematic information sharing for COVID-19-related signals and events was put in place among WHO country offices, Regional offices and headquarters.

**Figure 2:**
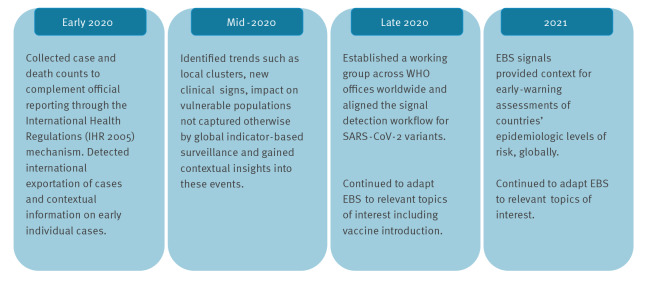
The adaptation of the World Health Organization’s public health intelligence activities for COVID-19, 2020–2021

COVID-19: coronavirus disease; PHI: public health intelligence; SARS-CoV-2: severe acute respiratory syndrome coronavirus 2; WHO: World Health Organization.

Since the first detection of COVID-19 cases, the processes and scope for COVID-19 EBS activities have evolved to reflect changing response priorities. During the early months of the pandemic, EBS complemented official COVID-19 case and death reporting according to the International Health Regulations (2005) [3] and triggered verification processes and responses. As the pandemic evolved, PHI activities evolved to provide a more comprehensive understanding of the pandemic that was not readily captured by one type of surveillance alone (Figure 2).

The scope of PHI evolved over time to include vaccine introduction, emergence of severe acute respiratory syndrome coronavirus 2 (SARS-CoV-2) variants, unusual clinical manifestations, upsurges in cases, hospitalisations and deaths at subnational level. Granular information collected through PHI provided valuable contextual information, which is not always available through IBS sources alone, including the burden on the health system and at-risk and vulnerable groups such as healthcare workers, rapid response teams, indigenous populations, children, pregnant women, elderly people and refugees.

The PHI approach combined signals detected via EBS and IBS along with contextual information which provided WHO response teams that supported the assessment and interpretation of COVID-19 epidemiological situations at national and subnational level. Analysts working on COVID-19 PHI activities validated and risk-assessed relevant signals, and communicated them through internal and external information products, including WHO Regional weekly briefing documents and the WHO’s weekly epidemiological update on COVID-19 [12]. In addition, PHI supported the interpretation of epidemiological trends collected through IBS which were shared publicly on the global COVID-19 dashboard [13], as well as other reports and dashboards.

**Figure 3:**
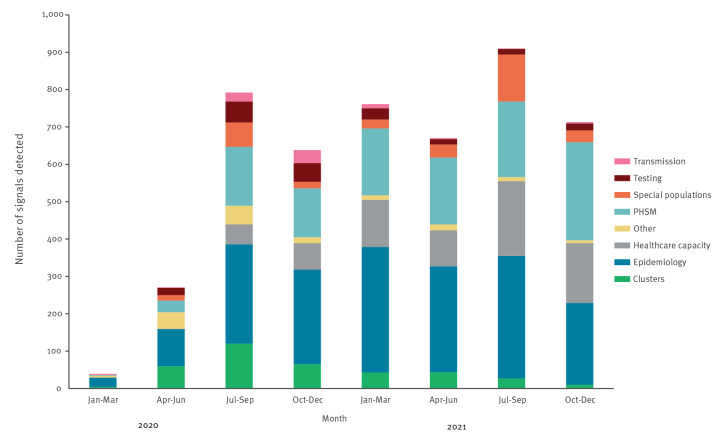
COVID-19 public health intelligence detected by the World Health Organization at global level by category type, as of December 2021

COVID-19: coronavirus disease; PHI: public health intelligence; PHSM: public health and social measures; WHO: World Health Organization.

Between 31 December 2019 and 31 December 2021, 4,794 COVID-19 signals were detected and followed up by the WHO at the global level using the EIOS system (Figure 3).

In May 2021, the WHO developed a situational alert system to support the early detection of countries where immediate action may help to mitigate the impact of a COVID-19 surge on morbidity and/or mortality. From May 2021 to June 2022, a collaborative, multi-disciplinary, mixed-methods process took place weekly, integrating information and input from teams across the three levels of the organisation. The first stage of the situational alert system employed an automated statistical risk assessment algorithm, based on daily incidence of cases and deaths, to predict COVID-19 disease severity within the near future. This produced an initial alert level for each country. The second stage integrated a PHI-based qualitative context assessment, which provided important information including pressure on the healthcare system; the impact of other concerning epidemiological signals such as concurrent outbreaks and large changes in circulating variants of concern; and the impact of factors affecting response activities such as acute events resulting in insecurity, or mass gatherings and population movement. This was combined with information on vaccination coverage, public health and social measures, and an assessment of the trust in available data for each indicator to produce a standardised recommendation for whether a country should be maintained at the initial alert level, or whether it should be updated. Based on this, teams at WHO global and regional levels jointly agreed on a final classification where the process supported a shared understanding of risk and operational priorities across the COVID-19 incident management teams at different levels of the organisation and, for specific contexts, the rapid release of response funds, distribution of oxygen, distribution of testing supplies, and technical support.

## Tailoring to Regional response needs

WHO was able to effectively adapt its PHI processes and systems including EIOS to align with surveillance strategies across different WHO offices as the pandemic evolved. An example of the unique adaptations implemented at the regional level was seen in the Western Pacific Region. Here, a media statement on cases of ‘unknown viral pneumonia’ in China (the first detection of COVID-19 cases) was detected through routine EBS processes on 31 December 2019 [14]. EIOS picked up a media report on this same signal in the Programme for Monitoring Emerging Diseases [15] on the same day. Subsequently, WHO rapidly established COVID-19-specific global surveillance activities to detect other related signals, focusing PHI to the needs and perceived risks within each Region. In the African Region, PHI was crucial in detecting signals of countries’ first cases and checking official statements for cases reported in the media. In the Region of the Americas, PHI was useful in detecting and contextualising outbreaks in cross-border areas, among cruise ship passengers and in congregate care settings and schools. The Eastern Mediterranean Region conducted PHI to detect upsurges in case or death incidence, track public health and social measures, particularly for schools, and later monitor vaccine roll-out. In the European Region, PHI activities focused on special populations and potential super-spreading events, and monitored subnational trends. The South-East Asia Region used PHI to monitor health systems capacities and reports of overwhelmed mortuaries and burial grounds during upsurges in the number of deaths.

Additionally, the WHO and partners conducted PHI for mass gathering events from December 2019 to December 2021, including the Union of European Football Associations championship that took place in 11 countries in Europe, the Tokyo Olympic and Paralympic Games in Japan, Hajj in Saudi Arabia, Al-Arbaeiin in Iraq and the World Handball Championship in Spain.

## Identifying strengths and weaknesses and addressing challenges

There are many strengths to the COVID-19 PHI activities implemented by WHO during the pandemic. WHO was able to develop a PHI approach throughout the course of the pandemic, used the findings to guide response activities and later to contextualise and supplement information derived from EBS and IBS as processes were established. The WHO’s PHI activities have remained flexible, scalable and resilient, adapting to the changing epidemiological context and availability of the WHO and partners’ person-time. Furthermore, the location of COVID-19 PHI activities within WHO’s Health Emergencies Programme and the Incident Management Support team structure was essential for coordinating and collaborating with key technical experts, ensuring relevancy and usefulness of information gathered, in order to better support Member States.

While the WHO’s experience with PHI during the COVID-19 pandemic illustrates its value for the timely detection of health threats [16] using multiple sources, there remain some limitations and challenges. Amid the rapid evolution of response needs, it was not possible to detect every potentially relevant piece of information across the range of topics of interest, particularly given the unprecedentedly high volume of information requiring triaging. This high volume of information necessitated an increase in the amount of person-time dedicated to COVID-19 PHI activities, while continuing to ensure sufficient resources for monitoring and detecting other public health events. Finding this balance with resource constraints proved challenging during several different phases of the pandemic. This suggests the need for continued improvement in response activities that enable public health intelligence teams to rapidly surge resources for PHI in future emergencies. While the evolving methods of PHI within the COVID-19 response are an operational strength, it also means that the type and volume of information varies throughout the course of a public health event WHO identified new technical challenges, including managing the different languages in which the information was written, their translation, identification of appropriate keywords for signal detection and the need for enhanced system support to improve signal detection. Signal detection could be improved by developing more targeted categories, and further addition of local or emerging key sources.

In response to these limitations and challenges, several initiatives have been developed to improve EBS activities for COVID-19 and other public health events. WHO offices established a COVID-19 PHI working group to strengthen collaboration, maximise resources and brainstorm solutions. From 31 March 2020 to 31 December 2021, the working group initiated a collaborative use of the EIOS system as one tool for detecting relevant information. The working group also provided regular feedback to further improve the process, including enhanced signal detection and verification, broadening of source types, geographical and language coverage and the use of tools to optimise shared workflows in rapid signal detection and monitoring. In addition to establishing the working group, WHO increased the number of personnel working on PHI, added and formalised trainings on PHI activities for COVID-19 and conducted a survey-based assessment of the system to further understand its strengths and weaknesses.

## Conclusion

Public health intelligence is a rapidly evolving approach that is necessary for WHO and Member States [17]. The COVID-19 pandemic presented a unique and challenging opportunity to develop PHI approaches for pandemic monitoring and response. WHO’s experience with PHI during the pandemic illustrate as the need for continuous development of PHI approaches for effective decision making to support Member States and inform response to a public health event. It also suggests that key future improvements should include greater automation tools that enable analysts to handle large quantities of information, additional training for personnel undertaking PHI and increased PHI capacity building within Member States and WHO. This would facilitate surge capacity in the future, further methodological development for systematic filtering of relevant information and establish evaluation processes for the PHI system. Building Member States and partners’ capacities to implement PHI approaches, in addition to IBS, for new and future public health threats has already begun, and it is important to continue developing these activities. Public health professionals and institutions dedicated to strengthening PHI capacity, including WHO’s recently established Hub for Pandemic and Epidemic Intelligence, can catalyse this change and build on the momentum created by the response to the COVID-19 pandemic.
